# Supplementary material for: Transposition-mediated DNA re-replication in maize
Source: eLife. 2014 Nov 18;3:e03724. doi: 10.7554/eLife.03724 (PMC4270019; doi:10.7554/eLife.03724)
Supplement: Supplementary file 1. — Sequences flanking the Composite Insertion in the TDDCI and solo-CI alleles and sequences flanking fAc in the deletion alleles. DOI: http://dx.doi.org/10.7554/eLife.03724.019 [file elife03724s001.docx]

**Supplementary file 1:**

Sequences flanking the Composite Insertion in the TDDCI and solo-CI alleles and sequences flanking *fAc* in the deletion alleles.

The color and orientation of the sequences are consistent with the color and orientation of the line segments in Fig. 1 and Fig. 2. The bold, underscored, and highlighted 8 bp sequence is the target site duplication.

**1. *P1-rr-T22/p1-ww-T22***

Distal junction of the CI in *P1-rr-T22*

GGCTGCTCGGCCACCGGGCCTTGAAGCGGCCCACCCAGCTGGTTTCTTTTAATAATAAGGTGAAAACACAAAATCGGACAGTTGTCTTCACTTCACCGGCGGGAGAACTACCTTTACACGCCAATCCATACGACTCTCTGCTCTCTATTCTACATGCCAGTACGTCATACACGAAACAGCACGTCAGCACGAGCTAAGAACAACATCTACAATAAAAAAAGAAAAAAAAACTGTACACACGAGCAACGCCTACTAGCGGCGTCGACCATATGGCCGGGCCACCTTGGTTGGTCTGTCTGTCTGTCTGATCGGTCCGATGACCGGAGCCGTCAGAACGAGTCGGACAGGAGCCAAGTCTCGAACGCCTCCAGCTCGCGATCGTCCGGGGTGCAGGCCGCCGTCGCAGCGGCAGCGGCAGCGGCAACAGCAGCCGCTTCCATCGGCTCGGCGGCCTGCCGCAGCTGCGCGCTGTGGTCGTCCTGCTCCGGCCCGCCCCACAGATGGGCCGCGAAGCCATCCCAGTCCATGTCGAACAGGTCGTCCACCTGGGCCTCGCCGCCGACCGCGCCAAGCCCTTGGAGGGCAGCCGAGTCGTGGCCGCCGGGCCCGATAGGCATCAGGGCGTCCATCTCGCTGTCGGCCTCCCCCCAGAGGTCCCCGAGCTCTATCGGCTCCAGCACCCACGGCCCGCTCGCGTCCTCGCTGCTGGGCCCTTCCTCCTCGGCCGTGCCCGTGCTGCCGCTGCTGCTGTTGGGCTGGTTCGGGCCCGGGTTCACC**GCGTCGCT**CAGGGATGAAAGTAGGATGGGAAAATCCCGTACCGACCGTTATCGTATAACCGATTTTGTTAgttTaTCCCGATCGaT

Proximal junction of the CI in *P1-rr-T22*

GTtTTCGTttCgtCCCGCAAGTTAAATATGAAAATGAAAACGGTAGAGGTATTTTACCGACCGTTACCGACCGTTTTCATCCCTA**GCGTCGCT**GTGCCGCGGGCTCGACGCCGCCGCGCCTTTCGCCTCGCCGCCGGGCTGATCGGCGTCCGAATGCTTGGTCCTGCTGGCGCTAGTCTTCGGCGGCCGGCCCGGGGTCCTGCCGCCGCGCCGCCTGTCGGCGCTCTGCAGCTTGCTCATGTCGATGGCGATGGCGGTATCGTCCGGCCCGGCGGTGTATTTCCGGCGGTACGTGTGGATCTGCCGGCTGAGGTGCGAGTTCCAGTAGTTCTTAATCT

*fAc* junction in *p1-ww-T22*

gTtTTCGTttcgtcccgcAAGTTAAATATGAAAATGAAAACGGTAGAGGTATTTTACCGACCGTTACCGACCGTTTTCATCCCTA**GCGTCGCT**GTGCCGCGGGCTCGACGCCGCCGCGCCTTTCGCCTCGCCGCCGGGCTGATCGGCGTCCGAATGCTTGGTCCTGCTGGCGCTAGTCTTCGGCGGCCGGCCCGGGGTCCTGCCGCCGCGCCGCCTGTCGGCGCTCTGCAGCTTGCTCATGTCGATGGCGATGGCGGTATCGTCCGGCCCGGCGGTGTATTTCCGGCGGTACGTGTGGATCTGCCGGCTGAGGTGCGAGTTCCaGTAGttct

**2. *P1-rr-E17***

Distal junction of the CI in *P1-rr-E17*

AtGATTTGACAGAACAGCCCGCACGATTTACATAGTTTAGAACAGAGACGCTACGACGGGAGGGGAGAGAACAGG**GGCACAGC**CAGGGATGAAAGTAGGATGGGAAAATCCCGTACCGACCGTTATCGTATAACCGATTTTGTTAGTTTaTCCCGATCGaT

Proximal junction of the CI in *P1-rr-E17*

CGTTTTCGTTTCCGTCCCGCAAGTTAAATATGAAAATGAAAACGGTAGAGGTATTTTACCGACCGTTACCGACCGTTTTCATCCCTA**GGCACAGC**CCCACGCTGCGACTGGAGAACTCGAGCGTATAGGTTCGAGTGTTTTTCTGGAGCCCAGAGATGCAGTATATTGAATGAATATTCTCTTTTTGGAGCCTATCGATTCTACGTAGAATCAAACCAAACACGCTATATACTATAATAGAGTGGCTCCATGACCCTATACTCTGGA

**3. *P1-rr-T24/p1-ww-T24***

Distal junction of the CI in *P1-rr-T24*

GGTCACGCCCATAATAAAACAATACATAATTTTAATATTTCACTACTACAATTTGTGAACGTATAGTATTTAAAAAATAATTTCCTGTTCACTATATTTCTTTTCACACATTTCGTCGAACATGTTGAATACATCGCTCGGGAGTTGTTATCTTCAAGCTCGTGGGCGAGCACAGGTGTTGCTCACAGTTCTTGCTCATGTCCATAGC**AGCATCGC**CAGGGATGAAAGTAGGATGGGAAAATCCCGTACCGACCGTTATCGTATAACCGATTTTGTTAGTTTTATCCCGATCGAT

Proximal junction of the CI in *P1-rr-T24*

TTACCGACCGTTTTCATCCCTA**AGCATCGC**TGGAGGTCTCCATCTTCGCTATACAAGCAGCAGAACTTGTCATCGCCGCACATGCCATGGTGCTCGACCAAACAGCCTAACCTCCAATCCTCCGCCATCTGCTTGTGGCAACTCGAGGTAGACCAAGTCATCACCGCCACGCCCGCCACCACCGTGCCTCCACAATCCGGCGACCCA

*fAc* junction in *p1-ww-T24*

ttaCcgaccgtTtTCatCCCTA**AGCATCGC**TGGAGGTCTCCATCTTCGCTATACAAGCAGCAGAACTTGTCATCGCCGCACATGCCATGGTGCTCGACCAAACAGCCTAACCTCCAATCCTCCGCCATCTGCTTGTGGCAACTCGAGGTAGACCAAGTCATCACCGCCACGCCCGCCACCACCGTGCCTCCACAATCCGGCGACCCATGTCTTCTGTTGTGCCTCGACTGAGCTGAAAGTGAAtGgatGAGGACGC

**4. *P1-rr-E340***

Distal junction of the CI in *P1-rr-E340*

AACCCGTCTCATCATCATCAGTGTAATAAGAGGCAAGGCAAAGGCAACAAGTACAGGCTACAAATCCCTTGCTTTCGTAGGTGCAGGCCAGGCCTTGCCCAGAGCTCCTCCCTCC**CCTCGCCC**CAGGGATGAAAGTAGGATGGGAAAATCCCGTACCGACCGTTATCGTATAACCGATTtTgTTAGTTTatCCCGATCGaT

Proximal junction of the CI in *P1-rr-E340*

CcgaccgtTTTCaTCCCTA**CCTCGCCC**CTCCCGCTGGAACGGAATGGAATCCGATGCGTGCCGTCACCATCATCGTTCCGTACAGGGCATCGTCGCTACATCAGCATCCGGGCGCTGATGGGGGTGTCTCCCTAGTAGTTTTAAAATGCCAATGTAATCAGCGTCTTTTTAGATGCGAAAGCCAGCCAGCTCCCATCCAGTTGAAGCGATGACGACGGGCGCAAGAGAAAGAAAAGCATGTATGCGTGCCAAATTAAAGCAAGGAGATGGAATTTTGCCAGCCCAACGAAAAGGCCATTGTGCGATTCAGCGggCTCATAAA

**5. *P1-rr-T21/p1-ww-T21***

Distal junction of the CI in *P1-rr-T21*

gtTTTGAGGACGGAGGAGGAGGTGTGAGCCGGTGAGACGTCGTCAGCCTGCCTGGGGCTCATCATGTGGGAAGCATGTGGTGGCTCTCCGCAAGCTGACAAGACAAGCGCGTGGTGGCACCACTCACTGGGCACTGCTGCAGCGGCGATTGGTCCCTCCCTCTCCCTGCAATTATTTGGCCATCTGAACGAAGACCCACGCGACGGCAGCGAGCGAGCAGTCGAGCACACACATATCTTGGGGGCTCTTGGATCGCCAAACTTGAATAATAATTGCGCAAGGGAGCAGTAGCCGACACATGGATGGCAAGACAAAGTGGGAGCAGGCAACAAACCACATAGGTTGGGGTTGGGCGATGGATCGAGCTCTCCACATAGGTTGAGGATACACAAGTCTTTTACTTGGAGTTGATCAA**GGTGCTGC**CAGGGATGAAAGTAGGATGGGAAAATCCCGTACCGACCGTTATCGTATAACCGATTTtgTTAgtttatccCGATcGat

Proximal junction of the CI in *P1-rr-T21*

GtTTTCGTTtcgTCCCGCAAGTTAAATATGAAAATGAAAACGGTAGAGGTATTTTACCGACCGTTACCGACCGTTTTCATCCCTA**GGTGCTGC**TTCAGTAGTTAAGAAGTTGCAGTTTGATGGATTGCCAAATAACGAGAATATCTGACAATCTAGATTTCAGACCGAAAAGACTACCAACTGGAAGAGAAGGAAAAGAGCATCGGA

*fAc* junction in *p1-ww-T21*

cccgtTtTCGTttcgtcccgcAAGTTAAATATGAAAATGAAAACGGTAGAGGTATTTTACCGACCGTTACCGACCGTTTTCATCCCTA**GGTGCTGC**TTCAGTAGTTAAGAAGTTGCAGTTTGATGGATTGCCAAATAACGAGAATATCTGACAATCTAGATTTCAGACCGAAAAGACTACCAACTGGAAGAGAAGGAAAAGAGCATCGGA

**6. *P1-rr-E311***

Distal junction of the CI in *P1-rr-E311*

TTCGTTCTCTGGTTGGTCGTCGTGCCCGTGCCAGCGCCGGCGTCCTCTTCTCCGTGCATGACGTGTGGGTCTGCTTCCGGTGGGCCCCGCCTCTGGATTACTGGCGTCTTCTCCTCAGAGGTTCTGTGCCT**ATTCGTCG**CAGGGATGAAAGTAGGATGGGAAAATCCCGTACCGACCGTTATCGTATAACCGATTttGTTAgtttatCCCGATCGat

Proximal junction of the CI in *P1-rr-E311*

cgttaccGAccGTTttcatcCCTA**ATTCGTCG**CCTCTCCGTAGAGAACAAGCTCTCGGTCCTCCTCTCTCATGGTCTCATTCTCATCTACGCCATCCGCCAGTGGTACTAATAACACAGTGTCACAGACTCAGAGTCGATAGATAAGTGAGAGTAGGGGGGCCTGGTCTAACTGATAGCATCGCAGGGCAGCACCGCGCTCTGCATGCATATGCCGCCATGCAGATGCCGAGGCAGGCATACAGCTGCTGCTGGCTGGACTGGCTCTGGCTGTTGCACCATGATGAGCTGAGACTGAGATACGCCACCATGTCCTGTATAAGTTGCGTTAATATCATATAAAACATCTTGTATGGTACCAAACCATAAGGCACACTACTTAATATAGTTTTTTAGTATTTACTTAAAAAATTGTATGGACTTGCCATACTGCTAAGTAAGGCATGTAGCGTGGCCTCGACACGCGCTCAGCTCTCGATCTGGATTCTAACCTTGTGGGTGGTGCCCGTTTGATTTGTGTATAAGTGTGTCACATTTTTACTAAGGTTAGTCGTTAGAATTGGATACCTAACCTTAGTAAAAAAATAGGCAAAGTGTATATGTCCGTCTCCATCCGTGTGGGCACGACGCC

**7. *P1-rr-E5***

Distal junction of the CI in *P1-rr-E5*

cCTCCCTGCAATTATTTGGCCATCTGAACGAAGACCCACGCGACGGCAGCGAGCGAGCAGTCGAGCACACACATATCTTGGGGGCTCTTGGATCGCCAAACTTGAATAATAATTGCGCAAGGGAGCAGTAGCCGACACATGGATGGCAAGACAAAGTGGGAGCAGGCAACAAACCACATAGGTTGGGGTTGGGCGATGGATCGAGCTCTCCTCATCGTCTGCCTTCCCCATTCCACAGCTCGCTTTCGGTCGAGCGAGCCTAATTCATCACAGACGCGCGCGGCATATATCTCTAGGCTAGATAGGCCGGCCGAGAATGTAATGTAACGGCGGCAAGGCAAATCGACTGACAGACAACATGGATGCATCGATCGATCTGACGCCGGGAACACTCCGAGAGCGAGACGTTTCGACTGGGAAGCCTGATGCTGATGCCTGACGGAGCACGCGGCGACTGTTTTGGCTGCC**GCGGACAC**CAGGGATGAAAGTAGGATGGGAAAATCCCGTACCGACCGTTATCGTATAACCGATTTTGTTAgtttaTCCCGATCGaT

Proximal junction of the CI in *P1-rr-E5*

ccgTTTTCGTTTCCGTCCCGCAAGTTAAATATGAAAATGAAAACGGTAGAGGTATTTTACCGACCGTTACCGACCGTTTTCATCCCTA**GCGGACAC**TGACTGGCCACCGAACCTTACGCGGACCGGAATACAAGGCAGGCAGTTGACAGTTCGCAGTTGGGTTGGGCCGGCATGGAGCCGGGCCACTGGCGGTGTGCAGTGCTGCCGAGCGAGACCTGACGACGGGAGAACGTGCTCGGGCAGAGGCGTCGTCGCTTCGAATGCCCCCTCCTTAGTCCTTCCTTCATTTTTGCCCGGCGTCGTGTGTCCCGACCAGACCTGCTGCGCTGGTGCTTGATATGATATGATCGTCAGGCAATACGTGAAGTGCTTGGTGAATGGTTAGGTTATCCACGCCGACGACGAAAGCGTCGGGCGACATGCACTGTGAGTCCCGCTGAGCAGAGCTTATCAGGTTGTACGCAGTACACGGTACTGACTGGCCTTTTCCGCGTCGACACGAAAACCATGTGCCATTGCTTGCTGTCGGCTTCTATCCCCTTGGTTGAAGGAGGAATGGGTCTCACTATGCCCACGAGCGCAGCGCTGATGGGTGCTTGTTGTAACAGTTGGAGTAGCAAGTGCCTAGCAGAGCAgtgaaaggTTCGGCG
